# (Co)Creating Cultures of Good Treatment in Health Education: What Actions Does the Community Propose?

**DOI:** 10.3390/bs16071211

**Published:** 2026-07-17

**Authors:** Javiera Ortega-Bastidas, Nancy Bastías-Vega, Marjorie Baquedano-Rodríguez, Cristhian Pérez-Villalobos, José Peralta-Camposano, Marcela Hechenleitner-Carvallo, Maritza Espinoza-Riffo, Angela Alarcón-Mella, María Isabel Ríos-Teillier, Alejandra Ricouz-Moya, Ricardo Arteaga-San Martín, Begoña Fierro-Antipi, Javiera Noriega-Aguilar

**Affiliations:** 1 Medical Educational Department, Faculty of Medicine, Concepción University, Chacabuco esquina Janequeo s/n., Concepción 4030000, Chile; nbastias@udec.cl (N.B.-V.); cperezv@udec.cl (C.P.-V.); mespinoza@udec.cl (M.E.-R.); analarcon@udec.cl (A.A.-M.); bfierro@udec.cl (B.F.-A.); jnoriega2018@udec.cl (J.N.-A.); 2 Department of Economics and Finance, Faculty of Business Sciences, Universidad del Bío-Bío, Avenida Collao 1202, Concepción 4030000, Chile; mbaquedano@ubiobio.cl; 3 Department of Health Sciences Education, Faculty of Medicine, Universidad de Chile, Avenida Independencia 1027, Santiago 8380000, Chile; jperalta@uchile.cl; 4 Department of Basic and Morphological Sciences, Office of Health Sciences Education, Faculty of Medicine, Universidad Católica de la Santísima Concepción, Alonso de Ribera 2850, Concepción 4090541, Chile; marcelahc@ucsc.cl; 5 Department of Clinical, Faculty of Medicine, Universidad Católica del Norte, Larrondo 1281, Coquimbo 1780000, Chile; mrios@ucn.cl; 6 Department of Health, Universidad de Los Lagos, Avenida Alberto Hertha Fuchslocher 1305, Osorno 5290000, Chile; alejandra.ricouz@ulagos.cl; 7 Institute of Movement Sciences and Human Occupation, Faculty of Medicine, Universidad Austral de Chile, Independencia 631, Valdivia 5110566, Chile; ricardo.arteaga@uach.cl

**Keywords:** health professions education, educational environment, qualitative action research, good treatment

## Abstract

Background: Educational environments grounded in respect, empathy, and fair treatment are vital for effective learning and clinical safety. Cultivating this positive culture is essential for improving professional practices and future development. The aim was to develop strategies collaboratively to promote good treatment practices within health programs across six Chilean universities. Methods: This study utilized a qualitative action research (AR) approach. Researchers conducted 23 co-creation workshops involving 188 students and 158 faculty members. The resulting data were analyzed using CAQDAS MAXQDA, version 24. Results: Participants favored short-term actions (61.6%; *n* = 94) over medium-term (17%; *n* = 26) or long-term (20.9%; *n* = 32) initiatives. Proposed short-term actions focused on curricular changes, faculty development, and well-being initiatives. Medium-term plans emphasized support systems and committees, while long-term goals targeted broader policies, labor conditions, and teaching hospitals, which presented the lowest frequency. The study identified three domains of actors involved: beneficiaries, groups, and those responsible for implementation. Conclusions: These community-driven, collaborative efforts provide a constructive framework for institutional intervention. By engaging stakeholders directly, the co-creation process allows for a comprehensive understanding of the scope and limitations of educational initiatives, effectively grounding them within the specific needs of the academic context.

## 1. Introduction

Why is it necessary to constantly rethink health training spaces? At times, the cultural norms and values of society guide educational agendas, impacting the curriculum, teaching, available resources and budgets, as well as the way in which formative quality is conceived (Crawford, 2025). At other times, the challenges of each era lead to questions about the new competencies that should be strengthened in the training of health professionals, as has been the case with the pandemic and the incorporation of artificial intelligence in health (Costa & Carvalho-Filho, 2020; Palés-Argullós, 2026). In this sense, rethinking health training spaces is a continuous exercise that must be safeguarded against the vortex of the avant-garde and the prevailing political guidelines. In line with this desire to keep educational reflection alive, the idea of “good treatment” emerges as a return to the principles of training. It is not a conceptual field that has required deep exploration, as it inherently denotes a fundamental character that needs to be recovered.

Under this understanding, it should not be forgotten that positive training environments reduce students’ errors in clinical simulations and real practice, because of the treatment received during training (Suikkala et al., 2021). Good treatment (buen trato) is considered—in the view of Dois and Bravo (2024)—to be a fundamental dimension of the clinical relationship, from which the right to health and respect for the other are intertwined, a condition that could hardly be learned outside the context of fair and empathetic assessment. As an emerging research phenomenon spearheaded largely in the Chilean context, buen trato (good treatment) addresses a critical empirical vacuum in health sciences education that remains widely under-explored. While this vacuum is frequently approached through established conceptual lenses such as educational environment, hidden curriculum, well-being, or humanization, *buen trato* offers a reflective pathway of return. This is because all these dimensions inherently rely on an a priori relational character that is intrinsically oriented toward the idea of the Good. What the scarce discussion around *good treatment* (buen trato) has managed to recover is, precisely, this foundational horizon. It is well known that this idea has been extensively problematized in philosophy, and one point on which philosophers agree is that the Good does not require grounding in the way that other ideas do. For Grondin (2015), the link between science and grounding is ancient; however, it was the Greeks who first distinguished those forms of evidence that, in principle, underlie all others when speaking of a fundamental idea. Based on this premise, this philosophical foundation is far from detached from empirical reality; rather, the idea of the Good acts as a fundamental ontological principle that can be purposefully channeled back into empirical discussion. Consequently, the present research has been guided by the notion of good treatment to highlight a dimension sometimes overlooked in scientific debate—particularly when engaging with concepts that do require grounding, such as educational environment, well-being, humanization, and educational culture—thereby reclaiming the essential question of the what as a vital imperative for our time.

In other words, rethinking health training sometimes requires only a return of focus to recover the meaning that underlies the principle of respect and patients’ rights in care (Abdullah & Fakieh, 2020). Therein lies the primary focus of a fundamental principle such as the Good, which constitutes the field of problematization that *good treatment* can offer as the preeminent pathway to (co)create training spaces that are responsive to local realities and the demands of the current era. (Co)creation is an invitation to recover the relational character of educational reflection, which should not be centered on the perspective of one or another educational agent. This is reflected in interventions that seek to engage directly with the population. Pandit et al. (2025) demonstrated that the community-engaged learning (CEL) model contributes to the development of competencies such as empathy, leadership, and communication skills, thereby fostering the personal and professional development of students. Aljafri et al. (2025), for their part, found that a community-based education (CBE) program improves teaching and communication skills with patients, significantly increasing the latter’s health literacy.

Educational experiences such as those already mentioned set precedents for the current discussion, as they reclaim the meaning of institutions and the practices carried out within them. Under this understanding, the incorporation of good treatment as a formative principle, in articulation with the community-engaged learning model, allows for progress toward a more comprehensive training, where knowing, doing, and being are oriented toward the development of truly humanized care. That said, this research will present a pathway that will allow the phenomenon of good treatment to be visualized as a means through which actions can be guided to improve the educational environment and are, simply put, understood within the idea of the Good. It should be noted that good treatment occupies this role in the present research for two reasons: First, there is sufficient evidence indicating that students in health programs are mistreated during their training process, and it is imperative to promote collaborative spaces that break with an entrenched dynamic that has become embedded in training. Second, educational interventions that yield the best results in the contexts in which they are implemented emerge from and for the same subjects. Creating spaces for dialog around good treatment allows intervention initiatives to remain feasible over time. For these reasons, the objective of this research was to collaboratively develop strategies to promote good treatment practices in health programs.

### 1.1. From a Culture of Mistreatment to a Culture of Good Treatment: How Do We Rethink Educational Practices Conducive to Professional Training?

The scientific literature has been clear in its descriptions and understanding of educational cultures permeated by everyday mistreatment. The existence of mistreatment in health professions education isa well-documented phenomenon across several health disciplines, such as medicine (Pradhan et al., 2019; Olivares et al., 2021; Bastías-Vega et al., 2021; Pillado et al., 2023; Hayward et al., 2023; Omer et al., 2025), dentistry (Rowland et al., 2010; Premadasa et al., 2011), midwifery (Shapiro et al., 2018; Capper et al., 2021), and nursing (Minton & Birks, 2019). In these contexts, mistreatment has consistently been shown to range from verbal and psychological abuse, humiliation, discrimination, and direct harassment (Munayco-Guillén et al., 2016; Barry & Shahbaz, 2025) to bullying, racism, and prejudice related to sexual orientation, overweight, or obesity (Mejía et al., 2018; Cormack et al., 2024).

Mistreatment is not an issue that occurs at a single moment in educational training, and it would be misleading to consider it in isolation, since its consequences indicate otherwise; that is, these experiences occur throughout an entire training program. Evidence demonstrates serious consequences for students’ well-being and mental health, making them more likely to develop high levels of burnout and lower levels of empathy compared with students who have positive learning experiences (Dyrbye et al., 2021), as well as a greater risk of dropping out due to the context (Cormack et al., 2024). Favorable clinical learning environments are essential, as exposure to disrespectful care practices leads to a poor understanding of clinical care (Huang et al., 2025).

The persistence of mistreatment urgently requires that existing interventions, such as faculty development programs or institutional anti-harassment policies, be effective (Kloos et al., 2023; Cook et al., 2025) and translate into concrete action plans (Lall et al., 2021). What remains striking is that mistreatment in training is rooted not only in hierarchical structures but also in culture (Hernández & Alvear, 2020; Barry & Shahbaz, 2025), and it is therefore not surprising that some faculty members have justified mistreatment as an inherent aspect of training (Bermeo et al., 2016). There is even evidence that fear of future mistreatment persists, as does its influence on perceptions of evaluation and possible professional options (Hayward et al., 2023).

Against this backdrop, there is an urgent need to transform educational spaces, as negative interpersonal interactions often occur individually (Vanstone et al., 2022). It is imperative to prioritize an inclusive culture that establishes transparent mechanisms within the educational process to eliminate bias and support professional development, while also addressing gender inequalities to ensure equitable opportunities (Omer et al., 2025). This implies fostering a culture of open dialog between students and faculty, as communication significantly influences the development of clinical practices (Daswani et al., 2025).

From this perspective, it is worth reiterating that positive educational spaces reduce students’ errors in clinical simulations and real practice due to the treatment they receive during training (Suikkala et al., 2021). According to Dois and Bravo (2024), good treatment constitutes the ideal foundation of clinical relationships, since it is linked to the right to health and respect for others. This condition could hardly be learned if it is not fostered through fair and empathetic assessment. Moreover, it involves semantic meanings such as “empathy”, “kindness”, “understanding”, “happiness”, “tolerance”, “solidarity”, “love”, “affection”, “help”, “support”, “communication” and “trust”, which, in summary, denote matters related to recognition and positive regard toward others (Ortega-Bastidas et al., 2024). Understood operationally, good treatment (buen trato) is defined in this study as the set of relational practices—encompassing curricular, faculty-development, and institutional dimensions—that educational actors in health programs identify as essential for creating environments of mutual respect, psychological safety, and professional growth. This empirically grounded working definition, derived from participants’ voices and the existing literature, constitutes the concrete referent through which the broader philosophical horizon of the Good is channeled into actionable educational strategies. In summary, the treatment received during training may have direct consequences for patient safety and the quality of clinical care (Suikkala et al., 2021).

Furthermore, evidence indicates a positive relationship between well-being and perceptions of the educational environment across different training experiences in the health field (Posada et al., 2023; Schwitz et al., 2023). Sattar et al. (2023), for example, have emphasized that positive mental health may reduce adverse outcomes associated with burnout. Moreover, professionals with compromised mental well-being experience difficulties in their work and sometimes hold an unfavorable view of their profession. Therefore, educational and work contexts that promote the well-being of their members are crucial for the sound development of professional practice. It is precisely the difficulties of daily life that permeate students’ well-being during training, and the urgency of addressing this lies not only in its usefulness for broadening the debate on well-being itself, but also in generating effective actions for cultural change (Schwitz et al., 2023) within educational institutions.

### 1.2. What Does It Mean to Promote a Culture Based on Good Treatment?

In recent years, a debate has emerged regarding how to safeguard the role of health professionals in the context of the integration of explainable artificial intelligence (XAI), which may increasingly permeate decisions about people’s care (Findik, 2026). In this context, the incorporation of AI could impact clinical practice, not only from an organizational perspective but also in the way professionals relate to their patients (Khosravi et al., 2024). Therefore, rethinking training spaces in parallel with a rapidly advancing discussion around technological innovation requires temporarily setting aside the conceptualizations that need to be grounded, to return to a fundamental idea such as good treatment. This was the primary intention of the line of research within which the results presented in this article are framed. Rather than merely focusing on the acquisition of technical-clinical skills, this field must aim for the internalization of a professional role, where the descriptive and relational meanings associated with good treatment are thoroughly explored; these terms are neither simple nor conceptually limited; rather, they carry a highly polysemous character for a phenomenon that has not been sufficiently addressed in the literature (Ortega-Bastidas et al., 2024). The ethical background of good treatment finds its ontological grounding in philosophy, an aspect that empirical approaches occasionally tend to bypass in mainstream conceptualizations. For this reason, the present study attempts to advance an empirical proposal for a notion that does not require the same foundational pathways as other phenomena in education.

In such a context, *good treatment* acquires fundamental relevance within a discussion that is driven by technological advances, but which risks depersonalizing health care if a return to fundamentally human principles is not made. The literature has addressed this issue carefully, as the treatment received during training may have direct consequences for patient safety and the quality of clinical care (Suikkala et al., 2021). What must not be forgotten is that rethinking and (co)creating health training spaces requires attending to the horizon from which one is being trained: the care of another person. To this end, not only must the curricular content be ensured, but also the very coexistence that unfolds within the educational training process. Moreover, university coexistence requires a declaration of principles, but also concrete actions through which a culture can be built that guarantees the desire to ‘be-together’, to form an educational community (Ortega et al., 2017).

In other words, health education must constantly rethink and interrogate training spaces to respond to the needs of future patients, but also of the educational actors who operate in that bridging scenario that exists between training and professional life. For this reason, it is essential to incorporate strategies that allow for the recognition and appreciation of social diversity, fostering the participation of all actors involved, particularly when these promote a dialog between different forms of knowledge (Fuentes-Vilugrón et al., 2025). The possibility of this occurring depends on educational environments characterized by respect, empathy, and fair treatment, as these conditions make it possible to foster the learning of theoretical knowledge and essential clinical skills (Merrick et al., 2021), reducing the risk of dropout and increasing subjective well-being (Hanco-Monroy et al., 2024). For Gauld et al. (2025), the validity of acquired knowledge depends on a culture that guarantees justice among clinicians and patients, as well as among clinicians themselves. Finally, coexistence and participation are dimensions of university life that can promote and transform social processes that impact local, national, and global challenges (Tobón et al., 2021). The challenge of all this is the co-creation of actions that emerge from different actors within the community, and this is precisely what the present research seeks to demonstrate.

## 2. Methods

### 2.1. Research Design

This study employed a qualitative action research (AR) design, operationalized through a co-creation methodology, to identify solutions for improving treatment in health education. The design drew on core principles from participatory action research (PAR) (Latorre, 2003), which was appropriate as a strategy for this study because it enables the reconstruction of practices and social discourses to promote good treatment, while also advancing a deeper understanding of such practices in university life as expressed in educational work. PAR is a research practice that enables the rethinking of contextual problems with and for participants, rather than merely conducting research about them (Murillo et al., 2011). Although AR and PAR originate from distinct disciplinary contexts, these two frameworks were integrated precisely because they share core socio-critical principles: the dissolution of the researcher-subject hierarchy, the positioning of participants as active epistemological agents, and a commitment to the collaborative co-creation of action strategies. Grounded in these shared principles, the design of the co-creation workshops was structured into a series of iterative activities that transitioned from personal to group analysis. This AR approach, operationalized through co-creation, was designed to foster a reflective space where participants could move from individual reflection toward a shared understanding of their reality, supporting the emergence of collectively grounded action strategies.

The research is part of a project funded by the National Agency for Research and Development (ANID), Chile, FONDECYT No. 1221913. As it is educational research, the guidelines provided by Chile’s National Regulations associated with scientific research involving people were followed: Law No. 19.628 and Law No. 20.120. Data collection was carried out after obtaining institutional authorization from each educational institution and informed consent in all cases.

### 2.2. Study Setting

The study was conducted among undergraduate health science students from 6 Chilean universities. This setting was chosen because it allows for a comprehensive examination of the experiences and perceptions of future healthcare professionals in a diverse range of academic environments. A total of 346 educational actors from health programs participated in this study: Physical Therapy, Speech and Language Therapy, Medical Technology, Midwifery and Childcare, and Medicine.

Of the total participants, 188 were students, of whom 34.4% (*n* = 65) were men and 65.6% (*n* = 123) were women. A total of 158 faculty members participated, of whom 27.9% (*n* = 44) were men and 72.1% (*n* = 114) were women.

### 2.3. Study Duration and Validation

The study was conducted over a three-year period, from 2023 to 2025. This timeframe was essential for the successful coordination of 23 co-creation workshops across six universities in Chile (public and private), representing diverse regions of the country. This extended duration ensured meaningful participant engagement and allowed for a thorough analysis, which significantly contributed to the robustness and validity of the research findings. The co-creation workshops were operationalized as follows. The workshops were differentiated by group (faculty members and students separately) to avoid encounters where the asymmetry of the interaction could interfere with the process.

Regarding data distribution and analytical density, the 23 co-creation workshops conducted across the participating health science programs yielded a total of 161 distinct Units of Analysis (UAs). This distribution across programs ensured a comprehensive mapping of the phenomenon, while the high volume of localized meaning units provided the empirical depth necessary to achieve thematic redundancy and ensure the traceability of the categorical matrix. The co-creation workshops were carried out, in some instances, with individual programs, and in others, through the integration of multiple healthcare careers. These variations occurred due to participant availability across the different universities. For this reason, the distribution of universities and programs is presented based on the UAs, as they accurately reflect the collaborative work generated within this setting. To safeguard institutional confidentiality, the distribution of universities is reported by geographical region.

The northern zone accounted for 8.6% (*n* = 14), the metropolitan zone for 11.8% (*n* = 19), the center-south zone for 62.1% (*n* = 100), and the southern zone for 17.3% (*n* = 28). Regarding the educational programs, the distribution was as follows: 60.2% (*n* = 97) corresponded to mixed-participation workshops involving multiple health careers; 12.4% (*n* = 20) to medicine; 9.9% (*n* = 16) to medical technology; 8.6% (*n* = 14) to physical therapy; 4.9% (*n* = 8) to obstetrics and childcare; and 3.7% (*n* = 6) to speech-language pathology. All the programs also participated in the mixed workshops, and no additional careers were included within the scope of this research.

### 2.4. Sample Size

Participants were selected through maximum variation sampling, seeking to ensure representation of the different programs and training levels within each university. The inclusion criterion for students was regular enrollment status, while those who had been absent for more than two months in each evaluated semester, regardless of the reason, were excluded. For faculty members, the inclusion criterion was responsibility for collaboration on any required, elective, or complementary course or training activity within each program’s curriculum; those who had been absent from their academic duties for the previous two months were excluded. This two-month exclusion criterion was established on the basis that the everyday life of the subject is a fundamental condition for activating in situ reflective processes. When a participant has been absent from the investigated context for two or more months, they may become disconnected from the current dynamics of academic life, which were essential for ensuring that the reflective process remained contingent upon and responsive to the lived realities of the training environment.

### 2.5. Data Collection Procedure

As a data production instrument, co-creation groups were conducted based on the conversation-on-paper technique and drawing on the principles of the “consciousness raising group” (CRG). Rooted in critical-participatory inquiry, the CRG is a methodological device through which participants progressively move from individual awareness of a shared experience toward collective critical reflection and the co-construction of transformative knowledge (Kamberelis & Dimitriadis, 2013). Unlike conventional focus groups, which primarily aim at generating descriptive accounts, the CRG is specifically designed to facilitate consciousness-raising as both a process and an outcome, making it particularly well-suited to PAR designs in which the goal is not only to understand but to collectively transform existing conditions (Kamberelis & Dimitriadis, 2013). In this study, the CRG framework was selected because it aligned with the emancipatory logic of PAR, enabling educational actors to articulate, examine, and collectively reframe their experiences of good treatment within their institutional contexts, particularly around the issue addressed in this research: proposed actions to strengthen an educational culture based on good treatment. The 23 co-creation workshops were conducted, with intra-group dynamics differentiated between student and faculty groups. Each workshop lasted approximately 90 min and typically involved around 12 participants.

First, institutional authorization was obtained from the 6 participating universities across Chile. Data collection was conducted only after receiving institutional approval and obtaining informed consent from all participants. These processes detailed the study’s objectives, potential uses, types of participation requested, associated risks, and guarantees of freedom and voluntariness. Participants were assured of the confidentiality of their involvement and informed of their right to withdraw at any time. Additionally, the process of anonymization was explained to participants in the co-creation workshops.

Each workshop was moderated by members of the research team, who guided the reflective process at each stage through open-ended questions and structured activities in which all participants took part. The first activity was individual, in which each participant was asked to identify behaviors characteristic of good treatment. In the second activity, participants were gathered into groups of no more than six people, where they were invited to reflect on what is currently being done well within their educational institution and what aspects should be improved. The third activity required participants to analyze the consequences that promoting a culture of good treatment would have on the training process. The fourth activity, entitled ‘Hands to Work’, asked participants to identify actions oriented toward good treatment and, finally, to present them in a plenary session to the entire workshop group. The reflective process across each of these activities was iterative, facilitating the participation of all participants in an environment of respect. Participants were empowered to make their own decisions in the process of selecting actions and identifying those responsible for carrying them out.

The initial activities served as preparatory steps to ensure that the subsequent reflective process would unfold autonomously, without direction from the moderators.

The workshops were not audio recorded. Data were gathered from the activities carried out on paper and systematized accordingly for analysis. This decision ensured that the reflective environment was not conditioned by the recording of the experience, an aspect that was highly valued by the participants.

The workshops followed a semi-structured facilitation guide based on the ‘conversation-on-paper’ technique, which adhered to a straightforward, collaborative structure. Each group developed their initial ideas on a flipchart, and upon completion of the activity, the flipcharts were exchanged with the adjacent group. This allowed participants to observe the reflections produced by their peers and contribute new ideas that further deepened the ongoing dialog before the flipcharts were subsequently returned to the original group. This activity was essential for the subsequent stages of the workshop, as it allowed reflection to move recursively from the individual to the collective level, and from the collective to a new way of engaging with the themes that brought the entire group together. Regarding research reflexivity, the coordinating team strictly maintained a position of external facilitation throughout this process, utilizing reflective journals to minimize academic hierarchy bias and ensure that the participants’ co-creative voices remained central to the discussion. These iterative cycles were deployed across all 23 workshops until data saturation was achieved; the criteria for reaching this threshold were established by our proposal criteria of density and authenticity (Ortega-Bastidas, 2020).

This structured progression ensured the implementation of the consciousness-raising framework, facilitating a reflective process that moved iteratively from personal awareness to collective critical reflection, ultimately informing the identification of concrete actions oriented toward good treatment within each institutional context.

### 2.6. Ethical Considerations

Participants were informed about the ethical aspects of the study through a detailed consent form. The study posed no anticipated physical risks; however, participants were informed that it might prompt reflections on their educational experience. Throughout the entire process, the objectives and subsequent uses of the study, the type of participation requested, the associated risks, and the guarantees of freedom and voluntariness, confidentiality of participation, and the right to withdraw at any time were explained in the greatest possible detail. The findings will be used to guide the design of university interventions to address these issues. All data provided by participants were secured through coded identification. Although participants’ IDs were initially collected for data organization, identifiers were replaced with unique codes before data storage to ensure anonymity. Only the lead researcher had access to the coding system, and participant identities were excluded from all analyses. Data were securely stored on password-protected devices, accessible solely by the research team and, if deemed necessary, by the reviewing Ethical-Scientific Committee.

### 2.7. Analysis Plan

For data analysis, the content analysis technique proposed by Bardin (1986) was used through three phases: (1) pre-analysis: the data were organized and prepared based on the documents obtained in the co-creation workshops; (2) material exploration: the documents were systematized into two categorical dimensions: co-created actions and the subjects involved in those actions; (3) treatment and interpretation of the results obtained: in accordance with the principles established by Bardin (1986), the meaning units (MUs) were grouped according to frequency and theoretical relevance, to define the final categorization. A total of 152 MUs were identified for the action dimension, and 471 for the actors-involved dimension. The CAQDAS MAXQDA (version 24) was used.

To ensure inter-rater reliability during data processing, two separate codebooks were employed, each subdivided into two areas: the action dimension and the actors-involved dimension. The data were initially processed using a pilot coding scheme that operated across two levels of analytical specificity: deductive and inductive. This scheme was structured based on a primary analysis that defined the meaning of each MU within its context of use. To establish consistency, the coding scheme was continually cross-examined by the research team during tracking meetings where analytical progress was reviewed. In these sessions, we established explicit conceptual boundary criteria for the recognition and description of both the MUs and the analytical Memos generated during data collection. This collaborative approach allowed us to select relevance criteria to determine how codes would be clustered into themes based on the underlying coding structure. Grounded in Saldaña’s (2016) framework, this iterative process followed a three-step approach: the first two steps established agreement on the codebooks so the four coders could achieve consistency in coding at different stages of data processing, while the third step effectively accounted for the possibility of conceptual refinement and change at the group level.

In accordance with Bardin’s postulates, only the ‘how’ can clarify the ‘why’. In this sense, the MUs correspond to the segment of content considered the base unit for the purposes of categorization and frequency counting. As previously described, the decomposition of this content analysis technique is organized into three phases. Among the various analytical decision options proposed by Bardin, the criteria of presence and frequency by summation were selected: the former as an indicator of meaning, and the latter as a recording unit that grows with frequency of appearance, as opposed to weighted frequency, intensity, contingency, or order criteria. In this way, Bardin’s content analysis is inductive by principle, while not being restricted to a deductive approach when the nature of the data so requires.

In light of the above, the following criteria of scientific rigor proposed by Lincoln and Guba (1985) were safeguarded: (a) credibility was ensured through investigator triangulation, whereby researchers agreed on common criteria for handling the qualitative data; (b) transferability was ensured through a detailed description of the data at each stage of the fieldwork process in the co-creation groups; (c) confirmability was addressed through detailed documentation of the data collection process, records, and analyses.

## 3. Results

Of the 346 educational actors who participated in this study, 45.7% (*n* = 158) were faculty members and 54.3% (*n* = 188) were students. The co-created actions aimed at promoting good treatment, along with the actors involved, are presented below.

### 3.1. Co-Creation of Time-Based Actions to Promote a Culture of Good Treatment

A total of 152 MUs were coded, forming the basis for categorizing actions aimed at promoting good treatment in Chilean health programs. These MUs were classified according to timeframe: (a) short term, 1 to 2 years; (b) medium term, 3 to 4 years; and (c) long term, 4 years or more. Overall, there was a clear tendency to prioritize short-term actions (61.6%; *n* = 94) over medium-term (17%; *n* = 26) and long-term (20,9%; *n* = 32) actions. Of the total number of proposed actions, 54.6% (*n* = 83) were identified by faculty members and 45.3% (*n* = 69) by students.

This pattern was consistent across both groups. In both cases, the priority given to short-term actions reflects a concern for interventions perceived as immediately feasible. Among faculty members, of the 83 proposed actions, 57.8% (*n* = 48) corresponded to short-term actions, 20.4% (*n* = 17) to medium-term actions, and 21.6% (*n* = 18) to long-term actions. As for the students, of the 69 identified actions, 66.7% (*n* = 46) focused on short-term actions, 13.0% (*n* = 9) on medium-term actions, and 20.2% (*n* = 14) on long-term actions.

From these actions, eight subcategories emerged according to timeframe, representing educational or institutional plans aimed at strengthening good treatment. As shown in Table 1, the largest concentration was in the short term, particularly in actions related to well-being (26.9%; *n* = 41), faculty development (23.6%; *n* = 36), and curriculum (11.1%; *n* = 17). In the medium term, actions were oriented toward implementing support systems (7.8%; *n* = 12) and well-being committees (9.2%; *n* = 14). In the long term, proposals pointed to broader institutional transformations, such as policies to promote good treatment (9.2%; *n* = 14), improved working conditions (6.5%; *n* = 10), and the creation of teaching hospitals (5.2%; *n* = 8).

Among the short-term actions, curriculum emerged as a pathway to strengthen comprehensive education. Participants proposed incorporating mandatory courses into health sciences education while protecting and further decentralizing activities commonly labeled as “extracurricular,” which were regarded as relevant educational spaces. From this perspective, the curriculum should address not only technical development, but also the construction of a broader professional identity. Furthermore, participants stressed that the specific roles and learning goals expected at each training cycle must be clearly communicated. The implementation of this transparency is highly feasible, given that Chilean health programs develop detailed curricular designs that must be formally presented to the National Accreditation Commission (CNA). To facilitate timely adjustments in relation to the graduate profile, they emphasized the importance of content-leveling processes across different curricular levels rather than solely at the beginning, as is common in Chilean universities—a measure that merely requires articulating the existing curricular frameworks within each program. In this line, participants proposed strengthening the role of the tutor-teacher, a figure that is already established in some schools.

Faculty development also remained a priority for educational improvement. Although courses on teaching methods, assessment, and planning continued to be regarded as important, participants incorporated new demands linked to humanized treatment as a central component of training. In this context, they highlighted areas including effective communication, neurodiversity, collaboration, sign language, and psychological safety. Beyond these areas, participants emphasized the importance of training faculty in the humanities, creating interdisciplinary spaces for collaboration among educators that extend beyond academic research. This could be operationalized through cross-sectoral, multi-stakeholder workshops, which are already common practices in some Chilean health programs. Consistent with this, they proposed creating opportunities for coordination and feedback across groups, strengthening interdisciplinary work between faculty members and students, and explicitly reinforcing the need for continuous support and effective feedback for clinical faculty within clinical training environments.

Regarding well-being, proposals were organized around three main areas. The first involved rethinking faculty support to promote positive mental health, emotional self-regulation, and reduced academic overload, while respecting students’ time at each stage of training. The second concerned the need to establish conflict mediation protocols, effective responses to abuse, harassment, or mistreatment, and good-treatment guidelines consistent with each university’s institutional principles. The third focused on recovering or creating physical spaces that would ensure rest and shared community life, thereby strengthening not only professional education but also university life more broadly.

When educational actors disaggregated these actions, well-being accounted for the highest proportion of short-term proposals (43.6%; *n* = 41), followed by faculty development (38.3%; *n* = 36) and curriculum (18.0%; *n* = 17). Although some differences were observed between students and faculty members, particularly in curriculum and faculty development, these were not substantial, suggesting a relatively shared prioritization of immediate actions (Table 2).

In the medium term, actions concentrated on two areas. First, participants proposed implementing formal support systems for students and faculty members (46.1%; *n* = 12). For students, this involved support strategies to complete internships, consideration of clinical learning styles, emotional self-regulation, and, in some cases, greater curricular flexibility. For faculty members, the proposal focused on guided training systems in clinical placements to support students comprehensively and communicatively, as well as on teaching strategies tailored to the specific educational settings in which instruction occurs. To maximize their impact, these development courses must align seamlessly with current curricular designs, maintaining the flexibility needed to address emerging, real-world contingencies while streamlining training processes. Furthermore, this formal support should foster a positive relationship between faculty members and students—for example, through better coordination of assessments, more effective communication with academic leaders, and greater consideration of faculty evaluations in decision-making—while actively promoting inter-university exchange programs to enrich the educational culture.

Second, 53.8% (*n* = 14) of medium-term actions involved the creation of committees. These would include a mistreatment policy that explicitly establishes the review of cases involving allegations, not only those in which a formal complaint has been filed. In extreme situations, participants even suggested considering student requests for faculty dismissal. They also emphasized the importance of establishing a centralized unit for good treatment, capable of designing a framework for coexistence within the university to prevent harmful educational environments, promote mutual participation, strengthen ties among health programs within the same faculty, and ensure that academic activities are conducted during working hours. By comparison, medium-term actions showed a clearer difference between groups in support systems, with a higher proportion of these proposals coming from faculty members (Table 2).

In the long term, proposals were oriented towards more structural institutional transformations. A total of 43.7% (*n* = 14) corresponded to policies to promote good treatment in health programs, including recurring working sessions on self-care for students and academic staff, as well as protected spaces for developing empathy, assertiveness, and dialog among educational actors. In the same vein, participants proposed creating discussion forums between faculty members and students to address institutional conflict resolution in concrete terms, both within and across groups.

Likewise, 31.2% (*n* = 10) of long-term actions focused on redefining working conditions to promote greater satisfaction and academic commitment. Participants pointed to the need for contractual hours sufficient to meet the academic workload effectively, including not only teaching, but also administrative management, research, and community engagement. They also suggested improving institutional systems of academic evaluation in ways consistent with gender-sensitive and inclusive perspectives. Finally, they proposed strengthening campus safety conditions so that universities may function as protected spaces for all actors involved and contribute positively to university life.

Lastly, it is worth noting that among all the subcategories analyzed in this study, long-term actions presented the lowest frequency, yet they remain empirically fundamental to the phenomenon under investigation. Within this scope, 25% (*n* = 8) of these actions reflected concern about the construction of high-complexity teaching hospitals for clinical simulation intended to support the different stages of health education and aligned with new Chilean legal regulations establishing rights and duties in both health and education. Crucially, simulation centers do not share the same characteristics as traditional hospitals, precisely because they lack the real-time clinical contingencies inherent to actual healthcare settings; therefore, increasing their complexity would directly foster the creation of spaces for situated clinical learning. In this area, a particularly pronounced difference was observed between groups, with faculty members referring to this proposal more frequently than students did. Differences were also observed in policies to promote good treatment, which students mentioned more often, and in working conditions, which faculty members highlighted more often (Table 2).

### 3.2. Actors Involved in Actions Promoting Good Treatment in Health Programs

A total of 471 MUs were coded, forming the basis for categorizing the actors involved in actions co-created by students and faculty members to promote a culture of good treatment in health programs in Chile. These units were organized by the timeframe of the actions (short-term, medium-term, and long-term), and, on that basis, three subcategories were identified: beneficiaries, groups, and those responsible. Of the total coded MUs,51.4% (*n* = 242) were identified by faculty members and 48.6% (*n* = 229) by students.

Overall, short-term actions identified students as the main beneficiaries (55.0%; *n* = 77). By contrast, medium-term actions were more evenly distributed between students and faculty members, suggesting that the anticipated benefits may extend to other members of the educational community (16.4%; *n* = 23). Whereas long-term actions projected a broader impact on the university community, including administrative staff, professionals, and university authorities (28.5%; *n* = 40). These patterns are consistent with the temporal progression already described for the co-created actions, although slight variations were observed in the frequencies reported by participants (Table 3).

Regarding the groups involved, faculty members emerged as the primary actor in implementing short-term actions (49.7%; *n* = 92), an attribution they themselves emphasized. In medium-term actions, smaller differences were observed between groups (16.2%; *n* = 30), whereas in long-term actions (34.0%; *n* = 63), greater agreement was found around the role of university authorities, including rectors, vice-rectors, deans, department heads, program directors, and internal legal offices (Table 3).

As for those responsible for overseeing the implementation of the proposed actions, in the short term, the greatest responsibility fell on program directors and heads of departments or units related to these issues, such as gender or inclusion (55.4%; *n* = 81), with no marked differences between the frequencies reported by faculty members and students. In the medium term, responsibility shifted toward deans and program directors (19.8%; *n* = 29), with more noticeable differences between the two groups, faculty members assigning greater responsibility to these actors. In the long term, responsibility was attributed to the rectors’ offices and vice-rectorates (24.6%; *n* = 36), a trend that was again more strongly emphasized in faculty discourse (Table 3).

Taken together, these findings show that the actions proposed by the educational community span different time horizons and involve multiple institutional levels. Short-term actions were more strongly associated with immediate educational and relational conditions, whereas medium- and long-term actions were associated with more formal and structural changes. In turn, the actors identified as beneficiaries, groups involved, and those responsible suggest that promoting a culture of good treatment is not limited to individual initiative but requires coordinated action across the university community (Table 3).

## 4. Discussion

Reflection on good treatment is increasingly necessary insofar as it concerns a domain that underpins the work of the different educational actors. The (co)creation of an educational culture requires certain considerations and intentions that may permeate the internal dynamics of Chilean educational institutions and serve as an example to others. The results of this research point to potential educational transformations in the field of university coexistence. This phenomenon illustrates how individuals relate to one another within a shared educational endeavor. Coexistence should be oriented toward reflecting the educational goals of institutions, considering the subjective dimension that constitutes them, whether relational, communal, academic, or institutional in nature (Echeverría et al., 2021).

Ultimately, the practical strategies for *buen trato* (good treatment) co-created by the participants in this study are not mere administrative adjustments; rather, they represent an empirical manifestation of what Grondin (2015) conceptualizes as the non-grounded, foundational evidence of the Good. By demanding humanized faculty development, structural support, and responsiveness to clinical contingencies, both students and educators are actively reclaiming the relational core that should govern health education. In this sense, our empirical findings channel this ontological principle back into clinical reality, demonstrating that the question of the Good is not an abstract philosophical luxury, but a vital, situated necessity for resisting the technocratic pressures of contemporary healthcare environments. Consequently, fostering a culture of good treatment (*buen trato*) serves as the vehicle par excellence for positive role modeling among clinical educators. In health sciences education, professional identity is profoundly shaped by the informal and hidden curricula (Suikkala et al., 2021); students internalize relational habits by observing how faculty members interact with patients, peers, and the students themselves under stress. When faculty development successfully equips clinical teachers with effective feedback mechanisms and emotional self-regulation strategies, it reinforces their capacity to demonstrate positive role modeling even during high-pressure clinical crises (Sattar et al., 2023). This alignment between explicit ethical principles and real-world behavior not only strengthens the educational trajectory of the student but also fortifies healthcare contexts, creating institutional environments that are structurally and culturally more resilient when responding to systemic contingencies.

The results reveal percentage differences among the actions proposed in the short, medium, and long term. This pattern indicates that participants identified a greater number of actions in the short term. This pattern was consistent across both faculty and student groups, suggesting a shared recognition that certain problems in this domain require immediate attention and that the resources necessary for their implementation are, at least partially, already available within different Chilean educational institutions. Rather than reflecting mere institutional readiness, this tendency is interpretatively grounded in the participants’ own discourse, which repeatedly identified concrete and actionable changes as priorities. This finding is consistent with research on educational climate, which suggests that actors within educational institutions tend to prioritize interventions that are perceivable and feasible within their immediate organizational context (Merrick et al., 2021; Hanco-Monroy et al., 2024). Faculty development has increased considerably in recent decades; however, the focus today must be on implementing changes that promote excellence in future patient care (Omer et al., 2025; Daswani et al., 2025; Gauld et al., 2025).

Further research should examine how these educational practices are transferred, ensuring alignment not only with the curriculum but also with the beliefs and practices of the educators who support the transition from curricular design to clinical practice.

The actions proposed in the medium term are also noteworthy, since they reveal an institutional turning point. This means that a culture oriented toward good treatment cannot remain subject to the goodwill of educational actors alone. From the perspective of organizational change, this finding is significant: it suggests that participants recognize the need to move beyond individual dispositions toward the institutionalization of structural supports (Tobón et al., 2021). In this sense, the creation of institutional practices, such as support systems and well-being committees, deserves emphasis, as these can foster good treatment with the aim of safeguarding not only the educational process but, more broadly, university life. This shift from individual to institutional responsibility is consistent with literature on participatory institutional transformation, which emphasizes that sustainable change in higher education requires governance structures that formally embed values of care, respect, and well-being into organizational policy (Fuentes-Vilugrón et al., 2025). Long-term actions are no longer oriented toward a declaration of principles, but rather toward the management of concrete financial resources that ensure job stability for the educational community and opportunities for clinical training through training centers dedicated to these purposes, without relegating all that responsibility to hospital-based clinical centers.

The results of this study also highlight that educational responsibility does not rest solely with faculty members but also with institutional authorities, who are responsible for safeguarding educational processes. This finding speaks directly to questions of governance in higher education, where the distribution of responsibility across different institutional actors is a critical determinant of educational culture (Gauld et al., 2025). At its core, the educational environment is a fundamental dimension of the teaching-learning process and has been considered by some authors to be the central component of education (Tobón et al., 2021; Fuentes-Vilugrón et al., 2025). An environment conducive to learning requires not only adequate planning and the implementation of teaching strategies consistent with the training context, but also the establishment of positive faculty-student relationships. In this way, an educational environment that fosters meaningful learning spaces is strengthened by opportunities in which both faculty members and students reach agreements, thereby facilitating the acquisition of theoretical and practical competencies.

It is worth noting that while faculty members and students shared a few priorities, particularly in relation to short-term actions oriented toward respect and empathetic communication, certain divergences also emerged between the two groups. Faculty members tended to emphasize actions related to pedagogical structure and institutional support, whereas students more frequently identified relational and emotional dimensions of the educational environment as areas requiring immediate attention. These divergences are not merely descriptive; they reflect the asymmetrical positions that each group occupies within the institutional hierarchy and underscore the importance of co-creation as a methodological strategy for surfacing perspectives that might otherwise remain invisible within conventional top-down approaches to educational change. Thus, our study not only contributes to understanding these dynamics but also provides crucial evidence to inform the formulation of educational interventions and policies that ensure the training of ethical, empathetic, and effective health professionals.

In relation to the above, some limitations of this study should be mentioned. The generalizability of the findings may be limited, since the study focused on specific universities in Chile, which may not reflect the full diversity of experiences across different educational and cultural contexts nationwide. Social desirability bias may have shaped some responses, given the participatory and group-based nature of the workshop activities. A further limitation concerns the data record itself: the co-creation workshops were documented through flipchart notes and structured group syntheses rather than audio-recorded and individually transcribed statements. As a result, the analysis relies on coded meaning units rather than on individually attributable verbatim quotations, this restricts the possibility of including literal excerpts in the Results section that directly reproduce how each participant expressed their ideas. Furthermore, the findings are context-specific and may not be directly transferable to health education systems operating under different institutional, cultural, or governance frameworks. The absence of longitudinal evaluation constitutes an additional limitation, as the study does not allow for an assessment of whether the co-created actions were effectively implemented or produced sustained changes in educational culture over time. Finally, the dynamics inherent to co-created workshop settings, while methodologically valuable, may introduce limitations in terms of the depth and spontaneity of individual reflection, as group processes can sometimes constrain the expression of minority or dissenting perspectives.

## 5. Conclusions

Regardless of the pace of technological development in the current era, it is worth recognizing how contemporary demands drive change, and these forces do not always come from outside, but also from within educational institutions themselves. As discussed in the introduction to this manuscript, cultural norms, societal values, and the challenges of each historical moment continuously interrogate the spaces in which health professionals are trained (Crawford, 2025; Costa & Carvalho-Filho, 2020; Palés-Argullós, 2026). The incorporation of explainable artificial intelligence, the lessons drawn from the pandemic, and the growing recognition of students’ rights within academic institutions are all forces that are reshaping the educational landscape from within. In this context, rethinking health training spaces is not merely a response to external pressures, but a continuous and necessary exercise of institutional self-reflection.

The findings of this study underscore that integrating the humanities into faculty development and adapting to clinical contingencies are not merely complementary curricular updates, but foundational components of a robust relational ethics in health professions education. Good Treatment as an ethical dimension becomes particularly vital when confronting the unpredictable contingencies inherent to healthcare environments. While high-complexity simulation centers offer valuable, low-stakes spaces for situated learning, they inherently lack the real-time, chaotic pressures of actual clinical settings. Therefore, preparing educators to navigate these authentic healthcare contingencies through an ethical lens ensures that unpredictable clinical demands do not compromise the commitment to empathy and psychological safety.

Technological advances and population need require educational institutions to respond to local and international contexts. Ensuring a culture of good treatment in health programs underscores the need for guidelines that foster students’ well-being, professional competencies, and academic success. Co-creating spaces for dialog promotes university coexistence, which, in practical terms, requires making transparent those situations, positive or otherwise, that emerge in a context that is, by nature, highly diverse, demanding, and constantly changing. Indeed, coexistence is not an ideal exempt from difficulties or setbacks among those who inhabit the university environment.

To coexist with others in a context as distinctive as the educational one requires questioning what kind of “co-existential” bond should be strengthened in universities, so that institutions may effectively address the tensions and disputes that may arise throughout their history (Ortega et al., 2017). Educational culture can foster important social transformations, especially when it arises from within itself and respects each actor involved. This research intends to position good treatment as a foundational element of a culture that promotes safe learning environments and seeks to reclaim two interpretive pathways: on the one hand, it affirms the specific actions that good treatment entails in practice; on the other hand, it attempts to recognize an ethical principle at its foundation. We are speaking of the operationalization of humanization in health and thinking of good treatment as a pathway to ensure subjective well-being in professional education.

In summary, the present research demonstrates that the co-creation of strategies oriented toward good treatment is not only feasible within the institutional contexts studied but constitutes a meaningful and necessary form of educational transformation. Change does not always require extraordinary resources or sweeping reforms; sometimes, it begins with the recovery of a fundamental idea, one that, as argued throughout this manuscript, does not require grounding in the way that other concepts do, precisely because it is already embedded in the foundational principles of what it means to educate and to care for another human being.

## Figures and Tables

**Table 1:** Progression of actions within the framework of the (co)creation of a culture of good treatment, according to faculty members and students (*n* = 152).

	Short Term			Medium Term		Long Term		
	Curriculum	Faculty Development	Well-Being	Support	Well-Being Committee	Policies to Promote Good Treatment	Working Conditions	Teaching Hospital
Frequency	17	36	41	12	14	14	10	8
Percentage	11.1%	23.6%	26.9%	7.8%	9.2%	9.2%	6.5%	5.2%

**Table 2:** Frequency and percentage of actions according to timeframe and educational actor.

Time Frame	Action	Faculty Members	Students	Total
Short term	Curriculum	41.1% *n* = 7	58.8% *n* = 10	17
	Faculty development	55.5% *n* = 20	44.4% *n* = 16	36
	Well-being	51.2% *n* = 21	48.7% *n* = 20	41
Medium term	Support system	75.0% *n* = 9	25.0% *n* = 3	12
	Well-being committee	57.1% *n* = 8	42.8% *n* = 6	14
Long term	Policies to promote good treatment	35.7% *n* = 5	64.2% *n* = 9	14
	Working conditions	60.0% *n* = 6	40.0% *n* = 4	10
	Teaching hospitals	87.5% *n* = 7	12.5% *n* = 1	8

**Table 3:** Frequency of beneficiaries, groups, and those responsible according to timeframe, faculty members, and students.

Domain of Involved Actors	Short Term	Medium Term	Long Term
Beneficiaries	Faculty members: 36 Students: 41 Total: 77	Faculty members: 15 Students: 8 Total: 23	Faculty members: 20 Students: 20 Total: 40
Groups	Faculty members: 45 Students: 47 Total: 92	Faculty members: 19 Students: 11 Total: 30	Faculty members: 26 Students: 37 Total: 63
Those responsible	Faculty members: 39 Students: 42 Total: 81	Faculty members: 21 Students: 8 Total: 29	Faculty members: 21 Students: 15 Total: 36

## Data Availability

The datasets used and analyzed during the current study are available from the corresponding author upon reasonable request.

## References

[B1-behavsci-16-01211] Abdullah R., Fakieh B. (2020). Health care employees’ perceptions of the use of artificial intelligence applications: Survey study. Journal of Medical Internet Research.

[B2-behavsci-16-01211] Aljafri A., Abba P., Sedghi A., Conte A., Jerjes W. (2025). Evaluating the impact of community-based medical education on health literacy and patient empowerment in underserved populations: A pilot cohort study. Clinics and Practice.

[B3-behavsci-16-01211] Bardin L. (1986). Análisis de contenido.

[B4-behavsci-16-01211] Barry A., Shahbaz A. (2025). The challenges and opportunities clinical education in the context of psychological, educational and therapeutic dimensions in teaching hospital. BMC Medical Education.

[B5-behavsci-16-01211] Bastías-Vega N., Pérez-Villalobos C., Alvarado-Figueroa D., Schilling-Norman M., Espinoza-Riffo M., Parra-Ponce P., Matus-Betancourt O., Toirkens-Niklitschek J. (2021). Maltrato en el pregrado de la carrera de medicina. Revista Médica de Chile.

[B6-behavsci-16-01211] Bermeo J., Castaño-Castrillón J., López-Román A., Téllez D., Toro-Chica S. (2016). Abuso académico a estudiantes de pregrado por parte de docentes de los programas de medicina de manizales, colombia. Revista de la Facultad de Medicina.

[B7-behavsci-16-01211] Capper T., Muurlink O., Williamson M. (2021). Social culture and the bullying of midwifery students whilst on clinical placement: A qualitative descriptive exploration. Nurse Education in Practice.

[B8-behavsci-16-01211] Cook S. C., Barnes G. D., Berlacher K., Capers Q. I., Fradley M. G., Reardon L. C., Echols M. (2025). Experiences of lesbian, gay, bisexual, transgender, and queer cardiology physicians and fellows in training. JACC: Advances.

[B9-behavsci-16-01211] Cormack D., Gooder C., Jones R., Lacey C., Stanley J., Paine S., Curtis E., Harris R. (2024). Māori medical student and physician exposure to racism, discrimination, harassment, and bullying. JAMA Network Open.

[B10-behavsci-16-01211] Costa M., Carvalho-Filho M. (2020). Una nueva época para la educación médica después de la COVID-19. FEM.

[B11-behavsci-16-01211] Crawford R. (2025). Responding to the de-professionalisation of teaching: Empowering teachers to enhance their pedagogy through action research. Education Sciences.

[B12-behavsci-16-01211] Daswani S., Gorecki E., Mellon L. (2025). “You are not taught to think about the words you use and then it just perpetuates”: A qualitative examination of medical students’ perspectives of stigmatising language in healthcare. BMC Medical Education.

[B13-behavsci-16-01211] Dois A., Bravo P. (2024). Ejercicio docente y buen trato al usuario en el encuentro clínico de enfermería. Educación Médica.

[B14-behavsci-16-01211] Dyrbye L., Satele D., West C. (2021). Association of characteristics of the learning environment and us medical student burnout, empathy, and career regret. JAMA Network Open.

[B15-behavsci-16-01211] Echeverría R., De Lille Quintal M., Evia N., Carrillo C. (2021). Convivencia universitaria inclusiva, democrática y pacífica: De lo personal a lo institucional. Revista de Estudios y Experiencias en Educación.

[B16-behavsci-16-01211] Findik M. (2026). Research trends and ethical perspectives on explainable artificial intelligence in emergency medicine: A bibliometric analysis. Scandinavian Journal of Trauma, Resuscitation and Emergency Medicine.

[B17-behavsci-16-01211] Fuentes-Vilugrón G., Sandoval-Obando E., Landeros-Guzmán D., Pérez-Quinteros L. E., Arriagada-Hernández C., Caamaño-Navarrete F., Etchegaray-Pezo P., del Val Martín P., Jara-Tomckowiack L., Muñoz-Troncoso G., Muñoz-Troncoso F. (2025). Linking education, culture and community: A proposal for an intercultural educational triad. Education Sciences.

[B18-behavsci-16-01211] Gauld C., Nicolle B., Constant A., Gagné-Julie A. (2025). The role of clinicians in the looping effect: Epistemic injustices and looping breaks. Medicine, Health Care and Philosophy.

[B19-behavsci-16-01211] Grondin J. (2015). El sentido de la vida: Un ensayo filosófico.

[B20-behavsci-16-01211] Hanco-Monroy D., Caballero-Apaza L., Abarca-Fernández D., Castagnetto J., Condori-Cardoza F., De Lama R., Carhuancho-Aguilar J., Gutierrez S., Gonzales M., Berduzco N., Delgado R., San-Martín M., Vivanco L. (2024). Medical professionalism and its association with dropout intention in peruvian medical students during the COVID-19 pandemic. Behavioral Sciences.

[B21-behavsci-16-01211] Hayward L., Mott N., McKean E., Dossett L. (2023). Survey of student mistreatment experienced during the core clinical clerkships. The American Journal of Surgery.

[B22-behavsci-16-01211] Hernández H., Alvear G. (2020). Violencia en la formación médica. Revista de la Facultad de Medicina de la UNAM.

[B23-behavsci-16-01211] Huang J., Wang C., Fu Y., Yango R., Zhang M., Guo L., Gamble J., Creedy D. (2025). Effects of empathy on the perspectives of respectful and disrespectful maternity care among nursing and midwifery students in china: A cross-sectional study. Nurse Educ Today.

[B24-behavsci-16-01211] Kamberelis G., Dimitriadis G. (2013). Focus group: From structure interviews to collective conversations.

[B25-behavsci-16-01211] Khosravi M., Zare Z., Morteza S., Izadi R. (2024). Artificial intelligence and decision-making in healthcare: A thematic analysis of a systematic review of reviews. Health Services Research and Managerial Epidemiology.

[B26-behavsci-16-01211] Kloos J., Simon E., Sammarco A., El-Nashar S., Bazella C. (2023). Neglect as an undefined and overlooked aspect of medical student mistreatment: A systematic review of the literature. Medical Teacher.

[B27-behavsci-16-01211] Lall M., Bilimoria K., Lu D., Zhan T., Barton M., Hu Y., Beeson M., Adams J., Nelson L., Baren J. (2021). Prevalence of discrimination, abuse, and harassment in emergency medicine residency training in the US. JAMA Network Open.

[B28-behavsci-16-01211] Latorre A. (2003). Investigación acción.

[B29-behavsci-16-01211] Lincoln Y., Guba E. (1985). Naturalistic inquiry.

[B30-behavsci-16-01211] Mejía C., Quiñones-Laveriano D., Isela J., Aguirre-Valenzuela E., Heredia-Torres P., Miñan-Tapia A. (2018). Factores socioeducativos asociados a la percepción de maltrato en estudiantes de medicina peruanos. Educación Médica Superior.

[B31-behavsci-16-01211] Merrick D., Mbaki Y., Pratten M., Simpson T. (2021). Exploring wellbeing in first year medical students amidst a curriculum change. BMC Medical Education.

[B32-behavsci-16-01211] Minton C., Birks M. (2019). “You cannot escape it”: Bullying experiences of new zealand nursing students on clinical placement. Nurse Education Today.

[B33-behavsci-16-01211] Munayco-Guillén F., Cámara-Reyes A., Muñoz-Tafur J., Arroyo-Hernández H., Mejia C., Lem-Arce F., Miranda-Soberón U. (2016). Características del maltrato hacia estudiantes de medicina de una universidad pública del perú. Revista Peruana de Medicina Experimental y Salud Pública.

[B34-behavsci-16-01211] Murillo F., Rodríguez S., Herráiz N., Prieto M., Martínez M., Picazo M., Castro I., Bernal S. (2011). Investigación-acción. Métodos de investigación en educación especial.

[B35-behavsci-16-01211] Olivares S., Gómez J., Flores C., Castañeda A., Lizzeth M., Esperón R., Valdez-García J. (2021). Me preparo para prevenir la violencia y el acoso en estudiantes de medicina en méxico. Revista Investigación en Educación Médica.

[B36-behavsci-16-01211] Omer M., Linh T., Alhamdan A., Machetan K., Nistor-Gallos D., Moritz I., Rivera T., Kim D., Lawson A., Maurer K., Posti J. (2025). Gender disparities and their impact on the professional experiences of female. neurosurgery residents in germany: A cross-sectional survey. World Neurosurgery.

[B37-behavsci-16-01211] Ortega P., Merchán J., Domínguez J., Pérez G. (2017). La convivencia universitaria. Entre el peso y la levedad.

[B38-behavsci-16-01211] Ortega-Bastidas J. (2020). ¿Cómo saturamos los datos? una propuesta analítica desde y para la investigación cualitativa. Interciencia.

[B39-behavsci-16-01211] Ortega-Bastidas J., Baquedano-Rodríguez M., Bastías-Vega N., Pérez-Villalobos C., Schilling-Norman M. J., Parra-Ponce P., Martín R. A.-S., Hechenleitner-Carvallo M., Ríos-Teillier M. I., Paredes-Villarroel X., Peralta-Camposano J., Ricouz-Moya A., Soto-Faúndes C., Williams-Oyarce C. (2024). Natural semantic networks: The concept of mistreatment and good treatment in students of health careers. Behavioral Sciences.

[B40-behavsci-16-01211] Palés-Argullós J. (2026). La evaluación en educación médica en la era de la inteligencia artificial: Repensar la evaluación. FEM.

[B41-behavsci-16-01211] Pandit R., Essers R., Pennings H. (2025). Community-engaged learning within the medical curriculum: Evaluating learning outcomes and implementation challenges. International Medical Education.

[B42-behavsci-16-01211] Pillado E., Debbie R., Eng J., Chia M., Conway A., DiLosa K., Gomez-Sanchez C., Shaw P., Sheahan M., Bilimoria K., Hu Y., Coleman D. (2023). Defining sources and ramifications of mistreatment among female vascular surgery trainees. Journal of Vascular Surgery.

[B43-behavsci-16-01211] Posada M., Vargas V., Orrego C., Cataño C., Vásquez E., Restrepo D. (2023). Educational environment and mental wellbeing of medical and surgical postgraduate residents in medellin, colombia. Revista Colombiana de Psiquiatría.

[B44-behavsci-16-01211] Pradhan A., Buery-Joyner S., Page-Ramsey S., Bliss S., Craig L., Everett E., Forstein D., Graziano S., Hopkins L., McKenzie M., Morgan H., Hampton B. (2019). To the point: Undergraduate medical education learner mistreatment issues on the learning environment in the united states. American Journal of Obstetrics and Gynecology.

[B45-behavsci-16-01211] Premadasa I., Wanigasooriya N., Thalib L., Ellepola A. (2011). Harassment of newly admitted undergraduates by senior students in a faculty of dentistry in sri lanka. Medical Teacher.

[B46-behavsci-16-01211] Rowland M. L., Naidoo S., AbdulKadir R., Moraru R., Huang B., Pau A. (2010). Perceptions of intimidation and bullying in dental schools: A multi-national study. International Dental Journal.

[B47-behavsci-16-01211] Saldaña J. (2016). The coding manual for qualitative researchers.

[B48-behavsci-16-01211] Sattar K., Saiful M., Nor Arifin W., Azhar M., Zarawi M. (2023). A scoping review on the relationship between mental wellbeing and medical professionalism. Medical Education Online.

[B49-behavsci-16-01211] Schwitz F., Torti J., Lingard L. (2023). What about happiness? a clinical narrative review with implications for medical education. Perspective on Medical Education.

[B50-behavsci-16-01211] Shapiro J., Boyle M. J., McKenna L. (2018). Midwifery student reactions to workplace violence. Women and Birth.

[B51-behavsci-16-01211] Suikkala A., Timonen L., Leino-Kilpi H., Katajisto J., Strandell-Laine C. (2021). Healthcare student-patient relationship and the quality of the clinical learning environment—A cross-sectional study. BMC Medical Education.

[B52-behavsci-16-01211] Tobón F., López L., Montoya R. (2021). Percepciones sobre la participación activa y la convivencia en una comunidad universitaria. Estudios Socio-Jurídicos.

[B53-behavsci-16-01211] Vanstone M., Cavanagh A., Molinaro M., Connelly C., Bell A., Mountjoy M., Whyte R., Griegson L. (2022). How medical learners and educators decide what counts as mistreatment: A qualitative study. Medical Education.

